# Tuberculosis Control Activities Before and After Hurricane Sandy — Northeast and Mid-Atlantic States, 2012

**Published:** 2013-03-22

**Authors:** Joseph Burzynski, Jay K. Varma, Edwin Rodriquez, Margaret J. Oxtoby, Thomas Privett, Alstead Forbes, Dawn Tuckey, Donato Ruggiero, Terence Chorba

**Affiliations:** Div of Disease Control, New York City Dept of Health and Mental Hygiene; Bur of TB Control, New York State Dept of Health; Tuberculosis Program, New Jersey Dept of Health; Div of TB Elimination, National Center for HIV, STD, and TB Prevention, CDC

On October 29, 2012, Hurricane Sandy struck the U.S. northeast and mid-Atlantic seaboard; the effects of the storm extended to southeastern and midwestern states and to eastern Canada. At the time, 1,899 residents in the most affected areas were undergoing treatment for tuberculosis (TB) disease or infection. To ascertain the operational abilities of state and local TB programs during and after the storm and to determine whether lessons learned from a previous hurricane were effective in ensuring continuity of TB patient care, CDC interviewed staff members at all of the affected state and city TB control programs, including those in areas with power outages and flooded streets, tunnels, and subway lines. The interviews determined that continuity of care for TB patients in programs affected by Hurricane Sandy was better preserved than it had been during and after Hurricane Katrina in August 2005. This improvement might be attributed to 1) prepared-ness measures learned from Hurricane Katrina (e.g., preparing line lists of patients, providing patients with as-needed medications, and making back-up copies of patient records in advance of the storm) and 2) less widespread displacement of persons after Hurricane Sandy than occurred after Hurricane Katrina. Maintaining readiness among clinicians and TB control programs to respond to natural disasters remains essential to protecting public health and preserving TB patients’ continuity of care.

## TB Programs Most Affected

Hurricane Sandy traveled along the Atlantic coast, affecting 24 states from Florida to Maine and west to West Virginia, Michigan, and Wisconsin. Coming ashore near Atlantic City, New Jersey, Hurricane Sandy caused particularly severe damage in New Jersey and New York. Overall, the 15 most affected TB control programs were in the mid-Atlantic (Delaware, District of Columbia, Maryland, North Carolina, Pennsylvania, Virginia, and the cities of Baltimore and Philadelphia) and northeast (Connecticut, Massachusetts, New Hampshire, New Jersey, New York, Rhode Island, and New York City).

By 1 week after Hurricane Sandy made landfall, all TB control programs had resumed normal patient-care operations. At least 10 of the 15 programs had been closed for 2 days (including those serving New Jersey, New York state, and New York City), either because of preparations in anticipation of the storm or because of the direct effects of the storm. None reported any significant damage to TB program infrastructure or equipment. Immediately after the storm, all TB program employees were accounted for; unlike the experience after Hurricane Katrina (1), there was no loss or gain of TB patients as a result of Hurricane Sandy. At least two programs reported giving their patients medications in advance of the storm for self-administered therapy (SAT), including medications that would otherwise have been administered using directly observed therapy (DOT). Reportedly, all patients who were placed on SAT were returned to DOT within a week after the storm had passed.

After the storm, program consultants from CDC’s Division of Tuberculosis Elimination (DTBE) assessed the affected programs and determined that no special assistance from DTBE was needed. Similar to what was done in the aftermath of Hurricane Katrina, DTBE activated the DTBE help desk on November 2, 2012, to facilitate Hurricane Sandy–related communications among TB controllers and nurse consultants throughout the country. However, unlike the extensive and sophisticated chain of communications after Hurricane Katrina, no calls were received by the help desk before it was deactivated on November 15.

Overall, in the 15 most affected TB control jurisdictions (11 states, three cities, and the District of Columbia), a total of 1,899 patients (including those with verified cases of active disease, suspected disease, and those treated for latent TB infection [LTBI]) were being treated by the TB programs just before Hurricane Sandy struck. By November 12, 2012, all active disease TB patients from the affected areas had been located and, if still indicated, had resumed TB treatment on DOT; all patients treated for LTBI also were accounted for. One patient (an LTBI patient from New Jersey) initially was thought to be lost to follow-up. However, it was subsequently confirmed that this patient had completed her course of treatment on SAT. Therefore, all 1,899 patients under treatment before the storm remained under treatment afterward.

## New York City

During and after Hurricane Sandy, New York City Department of Health and Mental Hygiene (DOHMH) TB clinics remained open with only minimal reduction in service; hours were reduced on the afternoon that the storm arrived, and one clinic in Staten Island was closed for 1 day after the storm because no patients were scheduled for appointments that day. Initially, there were difficulties with testing specimens for TB because the public health laboratory was operating for several days on back-up generator power and had limited Internet connectivity, and because many hospital, commercial, and reference laboratories were not functional as a result of storm-related complications. The New York State Department of Health Laboratory provided short-term backup for transport and processing of some TB specimens.

What is already known on this topic?

State and local tuberculosis (TB) control programs plan for emergencies with the potential to result in mass displacement of patients and disruptions in access to diagnostic services.

What is added by this report?

The lessons learned from the experience of Hurricane Katrina in 2005 were applied successfully in maintaining preparedness and TB control activities for persons undergoing TB treatment after Hurricane Sandy. All of the 1,899 patients undergoing treatment in the 15 TB control program areas most affected were fully accounted for; unlike Hurricane Katrina, there was no loss or gain in the number of TB patients within programs as a result of Hurricane Sandy.

What are the implications for public health practice?

To address issues of natural disasters in an efficient, effective manner, TB control programs need to continue to conduct systematic planning that will enable timely response.

Because of flooding and loss of power, Bellevue Hospital had to evacuate all of its patients, and the New York City TB program immediately had to find another secure hospital ward for six TB patients in Bellevue’s detention unit. All six were safely transferred to Lincoln Hospital in the Bronx. Two of the patients were considered infectious; they were transferred to negative-pressure isolation rooms. Within a week of their transfer to the new facility, a physician from DOHMH went to Lincoln Hospital to visit the patients and to consult with the physicians involved in their care. Each patient was assigned a DOHMH staff worker for case management. An outpatient TB clinic also was affected by closure of Bellevue Hospital.

Within a week, DOHMH had contacted 26 of the 27 patients who previously had been receiving antituberculosis medications through the DOT program at Bellevue Hospital. As patients began running out of medications, all contacted patients were given appointments at DOHMH clinics. By November 16, a total of 24 of 27 patients had been evaluated at DOHMH clinics and continued with DOT, either at a DOHMH clinic, through the DOHMH field staff, or through DOT field staff members from Bellevue Hospital. During the approximately 1 month that Bellevue Hospital remained closed, four patients were discharged as having completed therapy. Four patients decided to continue treatment at DOHMH clinics, and the remaining 16 patients returned to Bellevue Hospital for further evaluation and treatment.

## New Jersey

After Hurricane Sandy struck, the state motor pool in Newark initially had no fuel; however, all vehicles including those used by the TB program were accounted for, and none were damaged. The Global Tuberculosis Institute at the University of Medicine and Dentistry of New Jersey (UMDNJ) in Newark and the Waymon C. Lattimore Practice that administers ambulatory-care services at University Hospital at UMDNJ reopened on November 5, 2012. Immediately after the storm, communication with the coastal areas in New Jersey was limited because phone lines and cell towers had been damaged or destroyed by the storm. Also, access to affected areas was restricted to those who could show proof of residency or property ownership.

Twelve active TB patients lived in the most severely affected areas of New Jersey, and these patients had been given their TB medications before the storm to conduct SAT. Two counties that did not provide TB medications to patients before the storm subsequently added the number of days that therapy had been missed to the end of the patients’ course of therapy.

### Editorial Note

Ensuring appropriate diagnosis, treatment, and prevention of TB is the responsibility of the National TB Control Program and of TB control programs in public health departments across the United States. This report describes the challenges faced by TB programs in affected jurisdictions when Hurricane Sandy disrupted normal operations. Standard treatment and cure of TB disease requires a multidrug regimen administered under DOT for at least 6 months (2). Recommended treatment for LTBI can be for 3 months using a new 12-dose regimen (3) or ≤9 months using older daily regimens (4). Despite the challenges, health department workers helped ensure continuity of treatment for TB disease or infection for the 1,899 patients in the 15 most affected TB control jurisdictions. Unlike the situation after Hurricane Katrina struck the U.S. Gulf Coast in 2005, causing displacement of 62 (48%) of the 130 New Orleans–area patients (1), none of the affected areas reported displacement of any TB patients after Hurricane Sandy.

In preparation for Hurricane Sandy, the TB programs in New York City, New York state, and New Jersey implemented measures in advance of the storm to ensure continuity of care, including 1) preparing line lists of patients who might be affected, 2) providing patients with medications to self-administer in the event DOT was interrupted, 3) providing patients with a list of phone numbers to reestablish contact with the health department if they were displaced and obtaining contact information for patients’ relatives and friends in other parts of the country, 4) making back-up copies of patient records for potential sharing with new jurisdictions, and 5) moving essential TB treatment supplies to safer areas. These activities reflected lessons that had been learned from the disruptions in patient care after the landfall of Hurricane Katrina in 2005 (1).

During an initial disaster response, the most urgent public health priorities are providing safe and adequate shelter, water, food, and sanitation. Also important are interventions to minimize potential spread of communicable diseases, including TB, because displaced persons congregate in shelters and resettle in new communities. A lesson learned from both Hurricane Katrina and Hurricane Sandy was that all TB control programs should consider planning for emergencies that might result in mass displacement of patients or in disruptions in access to laboratory or other diagnostic services, and in supply of medications.
